# The association between number of doctors per bed and readmission of elderly patients with pneumonia in South Korea

**DOI:** 10.1186/s12913-017-2352-7

**Published:** 2017-06-08

**Authors:** Joo Eun Lee, Tae Hyun Kim, Kyoung Hee Cho, Kyu-Tae Han, Eun-Cheol Park

**Affiliations:** 1 Department of Public Health, Yonsei University College of Medicine, Seoul, Republic of Korea; 2 Institute of Health Services Research, Yonsei University College of Medicine, Seoul, Republic of Korea; 3 Department of Hospital Administration, Graduate School of Public Health, Yonsei University, Seoul, Republic of Korea; 4 Department of Preventive Medicine, Yonsei University College of Medicine, 50 Yonsei-ro, Seodaemun-gu, Seoul, 120-752 Republic of Korea

**Keywords:** Readmission, Pneumonia, Number of doctors per bed

## Abstract

**Background:**

There is an urgent need to reduce readmission of patients with pneumonia and improve quality of care. To assess the association between hospital resources and quality of care, we examined the effect of number of doctors per bed on 30-day readmission and investigated the combined effect of number of doctors per bed and number of beds.

**Methods:**

We used nationwide cohort sample data of health insurance claims by the National Health Insurance Service (NHIS) from 2002 to 2013. Pneumonia admissions to acute care hospitals among 7446 inpatients older than 65 were examined. We conducted a multivariate Cox proportional hazard model to analyze the association between the number of doctors per bed and 30-day readmission, as well as that of pneumonia-specific 30-day readmission with the combined effects of number of doctors per bed and number of beds.

**Results:**

Overall, 1421 (19.1%) patients were readmitted within 30 days and 756 (11.2%) patients were readmitted for pneumonia within 30 days. Patients with pneumonia treated by very low or low number of doctors per bed showed higher readmission (pneumonia-specific readmission: hazard ratio [HR] = 1. 406, 95% confidence interval [CI] = 1.072–1.843 for low number of doctors per bed; all-cause readmissions: HR = 1.276, 95% CI = 1.026–1.587 for very low number of doctors per bed, and HR = 1.280, 95% CI = 1.064–1.540 for low number of doctors per bed).

**Conclusions:**

This empirical study showed that patients with pneumonia cared for in hospitals with more doctors were less likely to be readmitted. Pneumonia-specific 30-day readmission was also significantly associated with the combined effect of the number of doctors and the number of hospital beds.

## Background

Pneumonia is more common in older individuals and remains as the leading cause of death among the elderly worldwide. Older patients with pneumonia experience longer hospital stays and more comorbidities than young adults [1]. According to 2013 Statistics Korea data, pneumonia has risen from the twelfth to the sixth leading cause of death, with the death rate increasing from 5.7 to 21.4 per 100,000 over the past 10 years [2]. In addition, pneumonia has the highest rate of admission among people aged over 50 and there were 232,000 pneumonia inpatients in 2013 [2]. Low birth rate and the rapidly aging population are major reasons for the increase in pneumonia in South Korea. Pneumonia patients older than 65 cost 6.7-fold more than those aged 15 to 44, and 75% of associated medical expenses are reported to be for hospital admission in Korea [3].

Pneumonia is a common reason for readmission after discharge and pneumonia patients are especially vulnerable to readmission [4, 5]. Readmission due to pneumonia after discharge is also related to increased medical expenses and mortality [6]. Hospital readmissions are an indicator of poor health care quality [7–10]. According to a 2009 meta-analysis, urgent readmissions were often classified as avoidable, with the most commonly used investigation period being 30 days after discharge [11]. In South Korea, readmission rates for pneumonia and the seven diagnosis-related groups are indicators used to evaluate hospital programs. Similarly, the U.S. Centers for Medicare & Medicaid Services (CMS) publicly report 30-day hospital-wide readmission rates for acute myocardial infarction (AMI), heart failure, pneumonia, Chronic Obstructive Pulmonary Disease (COPD), stroke, and hip/knee arthroplasty [12]. CMS identified hospital avoidable readmissions as a major reason for excessive medical costs and has tried to reduce avoidable readmission to improve quality of care and decrease unnecessary medical expenses [13].

Human resources in hospital, especially the number of doctors, may affect healthcare outcomes [14]. In addition, the demand for health care has increased in Korea. Nevertheless, the number of doctors in South Korea (2.2 doctors per 1000) is still lower than the Organization for Economic Cooperation and Development (OECD) average (3.3 doctors per 1000) [15]. This imbalance may affect negatively patient outcomes including readmission. Therefore, this study focused on the number of doctors per bed and pneumonia readmission to examine the association between professional human resource and patient outcomes.

To our knowledge, most readmission studies have focused on patient demographic and disease-specific factors and have analyzed all-cause readmission as the study outcome without using nationwide data [16–19]. Readmission may be affected by both patient and hospital factors [20]. Although pneumonia is a common reason for readmission which is related to poor health care quality, few Korean studies on readmission risk factors have assessed nationwide data, especially for pneumonia-specific readmission [9]. We aimed to identify the effect of patient and hospital characteristics on 30-day pneumonia-specific readmission to assess quality of care using a nationwide sample of cohort clinical data. This study hypothesized that characteristics affecting pneumonia-related readmission would be different from those of pneumonia-unrelated readmission.

## Methods

### Data source and study sample

A retrospective cohort study was performed using National Health Insurance Service (NHIS) claims from 2002 to 2013. NHIS covers all Korean citizens, as it requires mandatory enrollment. The NHIS sample data includes about 1 million people as a representative sample of the Korean population. We retrospectively reviewed health insurance claims of patients older than 65 years who were hospitalized with pneumonia between 2003 and 2013. Several inclusion criteria were considered to select the study sample. First, only patients over 65 years were selected because the characteristics of pneumonia are different in younger individuals. Second, we included inpatients who were diagnosed with pneumonia via the principal diagnosis (except clinics or physician offices and long-term care hospitals). Third, to consider prior health care utilization, we used data starting from the second year (2003). Finally, after excluding the missing data, we identified 7446 pneumonia patients who met these criteria. The number of hospitals included in this study sample was 1223.

### Ethics

This study was approved by the Institutional Review Board of Graduate School of Public Health in Yonsei University for using data and study design (2014–202). And, this study was not included informed consents from the patients, because the patient’s information had been already anonymized and de-identified prior to analysis.

### Outcome measures

Our primary outcome variable was readmission within 30 days of hospital discharge from pneumonia. Discharge and readmission dates were obtained from medical statements. Readmission was classified as pneumonia-related, pneumonia-unrelated, and all-cause readmission. In the current study, readmission was defined as pneumonia-related readmission if pneumonia was the primary diagnosis for readmission. Readmission was classified as pneumonia-unrelated readmission if patients were readmitted for alternative medical problems. All-cause readmission was defined as readmission for any cause after discharge from pneumonia.

### Independent variable

The interesting variable in this study was the number of doctors per bed. The number of doctors per bed was calculated based on the total number of doctors and number of beds in each hospital. Then, we grouped the number of doctors per bed into quartiles (very low, low, high, or very high).

### Covariates

According to our literature review and NHIS data availability, we used covariates of four categories: (1) demographic and socioeconomic variables: sex, age, and income level; (2) prior health care utilization: length of stay (LOS) in last year, number of outpatient visits in the last year, number of emergency visits in the last year, and charges in last year, LOS of index hospitalization; (3) risk and comorbidity: admission pathway, septicemia/shock, cancer, dementia, heart failure, vascular or circulatory disease, chronic obstructive pulmonary disease (COPD), asthma, renal failure, and liver disease; (4) hospital characteristics: hospital classification, ownership, number of beds, and availability of magnetic resonance imaging (MRI).

### Analytical approach

Continuous variables were described as means and standard deviations and categorical variables were described as frequencies and percents. χ^2^ and t-tests were performed to evaluate differences in characteristics according to readmission. Kaplan-Meier survival curves were constructed for the number of doctors per bed and comparisons were made using log-rank tests. We used Cox proportional hazard frailty models using random effect at hospital level to examine the effect of patient and hospital characteristics on 30-day readmission for pneumonia. SAS 9.4 software (SAS Institute Inc., Cary, NC) was used for all analyses.

## Results

### Patient and hospital characteristics

The descriptive statistics stratified by 30-day pneumonia-specific readmission, 30-day pneumonia-unrelated readmission, and 30-day all-cause readmission are shown in Table 1. In total, 7446 subjects were analyzed. Of all patients hospitalized with pneumonia between 2003 and 2013, 756 (11.2%) were readmitted for pneumonia within 30 days numbered, 665 (9.9%) for other medical problems within 30 days, and 1421 (19.1%) for all causes within 30 days were. The average health care utilization in the prior year was higher in readmission groups than those not readmitted. Patients readmitted for pneumonia within 30 days had higher proportions of septicemia/shock, vascular or circulatory disease, COPD, asthma, and liver disease compared to those not readmitted. On the other hand, the patients readmitted for other medical problems or all causes within 30 days showed higher proportions of septicemia/shock, cancer, heart failure, vascular or circulatory disease, COPD, asthma, renal failure, and liver disease than those who were not readmitted.

**Table 1:** Characteristics according to 30-day pneumonia-specific, pneumonia-unrelated, and 30-day all-cause readmission rates among inpatients with pneumonia in 2003–2013

	No 30-day readmission	30-day pneumonia- specific readmission		30-day pneumonia-unrelated readmission				30-day all-cause readmission		
	*N*, Mean	N(%), Mean ± SD	*p*-value	N(%), Mean ± SD		*p*-value		*N*(%), Mean ± SD	*p*-value	
<Individual level>										
Sex										
Men	3101	405	(11.6)	0.276	377	(10.8)	0.012	782	(20.1)	0.017
Women	2924	351	(10.7)		288	(9.0)		639	(17.9)	
Age										
65–69	1020	104	(9.3)	0.092	108	(9.6)	0.014	212	(17.2)	0.047
70–74	1319	186	(12.4)		173	(11.6)		359	(21.4)	
75–79	1450	172	(10.6)		175	(10.8)		347	(19.3)	
80–84	1240	156	(11.2)		125	(9.2)		281	(18.5)	
≥ 85	996	138	(12.2)		84	(7.8)		222	(18.2)	
Income										
Medical aid	488	89	(14.3)	0.003	42	(7.9)	0.452	131	(21.2)	0.409
Low	1538	171	(9.1)		172	(10.1)		343	(18.2)	
Middle	1783	229	(10.3)		202	(10.2)		431	(19.5)	
High	2216	267	(9.8)		249	(10.1)		516	(18.9)	
Prior year LOS	52.1	59.6	±70.8	0.006	60.5	±60.4	0.001	60.1	±66.1	<.0001
Prior year number of primary care visits	30.0	32.4	±35.6	0.082	34.4	±36.0	0.003	33.4	±35.8	0.001
Prior year number of emergency department (ED) visits	0.4	0.7	±4.3	0.108	0.5	±1.5	0.059	0.6	±3.3	0.036
Prior year cost (1000won)	3055.8	4488.9	±11,796.2	0.001	3742.9	±6367.7	0.010	4138.0	±9635.6	<.0001
Pathway										
Outpatient	2262	269	(10.6)	0.312	241	(9.6)	0.537	510	(18.4)	0.259
Emergency	3763	487	(11.5)		424	(10.1)		911	(19.5)	
LOS of the index hospitalization	10.4	12.7	±131	0.144	10.8	±8.7	0.752	11.817	±11.3	0.415
Septicemia/shock										
Yes	155	31	(16.7)	0.021	28	(15.3)	0.020	59	(27.6)	0.002
Cancer										
Yes	452	65	(12.6)	0.319	138	(23.4)	<.0001	203	(31.0)	<.0001
Dementia										
Yes	243	28	(10.3)	0.736	35	(12.6)		63	(20.6)	
Heart failure										
Yes	221	29	(11.6)	0.898	51	(18.8)	<.0001	80	(26.6)	0.001
Vascular or circulatory disease										
Yes	440	73	(14.2)	0.026	94	(17.6)	<.0001	167	(27.5)	<.0001
COPD										
Yes	769	178	(18.8)	<.0001	170	(18.1)	<.0001	348	(31.2)	<.0001
Asthma										
Yes	796	198	(19.9)	<.0001	158	(16.6)	<.0001	356	(30.9)	<.0001
Renal failure										
Yes	182	28	(13.3)	0.363	40	(18.0)	<.0001	68	(27.2)	0.001
Liver disease										
Yes	152	32	(17.4)	0.009	31	(16.9)	0.002	63	(29.3)	0.000
<Hospital level>										
Hospital classification										
Teaching hospital or general hospital	4331	498	(10.3)	0.001	500	(10.4)	0.079	998	(18.7)	0.227
Hospital	1694	258	(13.2)		165	(8.9)		423	(20.0)	
Ownership										
Public	467	54	(10.4)	0.604	46	(9.0)	0.490	100	(17.6)	0.392
Private	5558	702	(11.2)		619	(10.0)		1321	(19.2)	
Number of beds										
Low	1645	252	(13.3)	<.0001	169	(9.3)	0.054	421	(20.4)	0.116
Middle	1747	245	(12.3)		173	(9.0)		418	(19.3)	
High	2633	259	(9.0)		323	(10.9)		582	(18.1)	
Number of physicians per bed										
Very low	1232	188	(13.2)	<.0001	134	(9.8)	0.269	322	(20.7)	0.002
Low	1960	307	(13.5)		205	(9.5)		512	(20.7)	
High	1208	119	(9.0)		129	(9.7)		248	(17.0)	
Very high	1625	142	(8.0)		197	(10.8)		339	(17.3)	
MRI										
Yes	5179	634	(10.9)	0.134	579	(10.1)	0.433	1213	(19.0)	0.591
Year										
2003	189	12	(6.0)	0.002	16	(7.8)	0.355	28	(12.9)	0.004
2004	262	19	(6.8)		32	(10.9)		51	(16.3)	
2005	338	51	(13.1)		32	(8.7)		83	(19.7)	
2006	393	33	(7.8)		38	(8.8)		71	(15.3)	
2007	421	44	(9.5)		46	(9.9)		90	(17.6)	
2008	471	69	(12.8)		59	(11.1)		128	(21.4)	
2009	526	57	(9.8)		45	(7.9)		102	(16.2)	
2010	543	78	(12.6)		68	(11.1)		146	(21.2)	
2011	855	120	(12.3)		86	(9.1)		206	(19.4)	
2012	997	148	(12.9)		130	(11.5)		278	(21.8)	
2013	1030	125	(10.8)		113	(9.9)		238	(18.8)	
Total	6025	756	(11.2)		665	(9.9)		1421	(19.1)	

*Abbreviations*: *COPD* chronic obstructive pulmonary disease, *ED* emergency department, *LOS* length of stay, *MRI* magnetic resonance imaging.

Foot note: n(%) for categorical variables; Mean ± SD for continuous variables

Hospital-level characteristics according to readmission among inpatients with pneumonia are shown in Table 1. Among patients hospitalized with pneumonia, those admitted to hospitals with higher number of beds were less likely to be readmitted for pneumonia or all-cause than those admitted to smaller hospitals. Patients admitted to hospitals with higher number of doctors per bed were also less likely to be experience pneumonia-specific or all-cause readmissions.

### Kaplan-Meier curves of 30-day readmissions for pneumonia according to number of doctors per bed

**Fig. 1:**
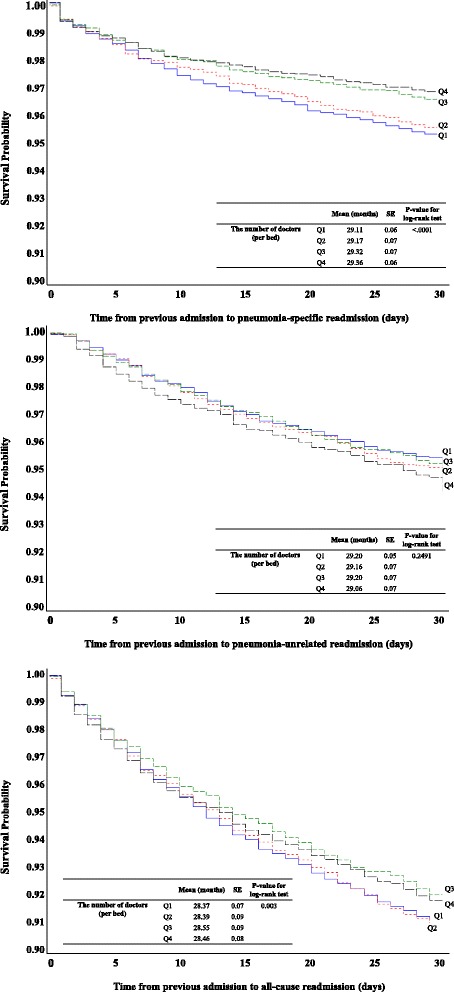
Kaplan-Meier curves of all-cause 30-day readmissions according to the number of doctors per bed

Kaplan-Meier curves of 30-day pneumonia-specific, pneumonia-unrelated, and all-cause readmissions according to the number of doctors per bed (Q1, Q2, Q3, and Q4) showed differences. This study defined six groups of pneumonia patients: (1) 30-day pneumonia-specific readmissions by the number of doctors per bed(13.5% readmission rate for Q1, 13.2% readmission rate for Q2, 8.97% readmission rate for Q3, and 8.04% readmission rate for Q4), (2) 30-day pneumonia-unrelated readmissions by the number of doctors per bed (9.81% readmission rate for Q1, 9.47% readmission rate for Q2, 9.65% readmission rate for Q3, and 10.81% readmission rate for Q4),, (3) 30-day all-cause readmissions by the number of doctors per bed(20.72% readmission rate for Q1, 20.71% readmission rate for Q2, 17.03% readmission rate for Q3, and 17.26% readmission rate for Q4), (Fig. 1). The log-rank test detected significant differences between very low, low, high and very high number of doctors per 100 beds for pneumonia-specific and all-cause readmissions (*p* < 0.0001 and *p* = 0.003), but not for pneumonia-unrelated readmission (*p* = 0.2491).

### Cox proportional hazard models of 30-day readmissions for pneumonia

In the multivariable analysis, the number of doctors per bed was significantly associated with pneumonia-specific, and all-cause readmissions (pneumonia-specific readmission: hazard ratio [HR] = 1.406, 95% confidence interval [CI] = 1.072–1.843 for Q2; all-cause readmissions: HR = 1.276, 95% CI = 1.026–1.587 for Q1, and HR = 1.280, 95% CI = 1.064–1.540 for Q2) (Table 2). LOS in the prior year showed a negative association with pneumonia-specific readmission (HR = 0.836, 95% CI = 0.710–0.983), but costs in the prior year showed a positive association with pneumonia-specific readmission (HR = 1.107, 95% CI = 1.015–1.206). LOS of the index hospitalization showed a positive association with pneumonia-specific, pneumonia-unrelated, and all-cause readmissions (HR = 1.250, 95% CI = 1.149–1.360; HR = 1.262, 95% CI = 1.162–1.370, and HR = 1.236, 95% CI = 1.165–1.311, respectively). In addition, asthma showed associations with higher readmission for pneumonia-specific, pneumonia-unrelated, and all-cause readmissions (HR = 1.211, 95% CI = 1.041–1.432; HR = 1.252, 95% CI = 1.070–1.466; and HR = 1.222, 95% CI = 1.093–1.367, respectively). About 1.03% between-hospital variation was observed in this study, in explaining the association between the number of doctors and pneumonia readmission.

**Table 2:** Multivariate Cox proportional hazard models with 30-day pneumonia-specific, 30-day pneumonia-unrelated, and 30-day all-cause readmission rates for pneumonia in 2003–2013

	30-day pneumonia-specific readmission			30-day pneumonia-unrelated readmission			30-day all-cause readmission		
	HR	95% CI		HR	95% CI		HR	95% CI	
<Individual level>									
Sex									
Men	1.000			1.000			1.000		
Women	1.010	0.875	1.166	1.072	0.930	1.235	1.040	0.940	1.150
Age									
65–69	1.000			1.000			1.000		
70–74	1.223	0.968	1.547	1.128	0.903	1.410	1.174	0.999	1.380
75–79	1.069	0.843	1.355	1.093	0.875	1.364	1.090	0.927	1.282
80–84	1.101	0.864	1.402	1.046	0.830	1.318	1.086	0.918	1.283
≥ 85	1.175	0.907	1.522	0.919	0.703	1.201	1.058	0.879	1.274
Income									
Medical aid	1.323	1.020	1.717	0.796	0.586	1.080	1.048	0.861	1.276
Low	1.037	0.864	1.245	1.089	0.916	1.294	1.064	0.938	1.205
Middle	1.031	0.868	1.223	0.965	0.817	1.141	0.993	0.881	1.118
High	1.000			1.000			1.000		
Prior year LOS	0.836	0.710	0.983	1.181	1.001	1.393	0.997	0.888	1.120
Prior year number of primary care visits	0.919	0.823	1.027	0.945	0.846	1.056	0.929	0.859	1.005
Prior year number of emergency department (ED) visits	1.008	0.990	1.025	0.995	0.965	1.025	1.003	0.988	1.019
Prior year cost (1000won)	1.107	1.015	1.206	0.904	0.828	0.986	1.001	0.942	1.064
Pathway									
Outpatient	1.000			1.000			1.000		
Emergency	1.113	0.959	1.291	1.002	0.866	1.161	1.053	0.949	1.168
LOS of the index hospitalization	1.250	1.149	1.360	1.262	1.162	1.370	1.236	1.165	1.311
Septicemia/shock									
No	1.000			1.000			1.000		
Yes	1.177	0.846	1.638	1.298	0.931	1.810	1.223	0.968	1.545
Cancer									
No	1.000			1.000			1.000		
Yes	0.540	0.424	0.687	1.212	1.015	1.447	0.877	0.762	1.009
Dementia									
No	1.000			1.000			1.000		
Yes	0.605	0.414	0.885	1.126	0.832	1.526	0.837	0.660	1.062
heart failure									
No	1.000			1.000			1.000		
Yes	0.695	0.492	0.980	1.181	0.904	1.544	0.930	0.754	1.148
Vascular or circulatory disease									
No	1.000			1.000			1.000		
Yes	0.749	0.584	0.961	1.155	0.938	1.422	0.968	0.826	1.134
COPD									
No	1.000			1.000			1.000		
Yes	0.897	0.760	1.059	1.171	1.003	1.367	1.029	0.919	1.152
Asthma									
No	1.000			1.000			1.000		
Yes	1.221	1.041	1.432	1.252	1.070	1.466	1.222	1.093	1.367
Renal failure									
No	1.000			1.000			1.000		
Yes	0.842	0.600	1.182	1.122	0.840	1.500	0.977	0.784	1.217
Liver disease									
No	1.000			1.000			1.000		
Yes	0.848	0.592	1.214	1.155	0.845	1.577	0.993	0.785	1.257
<Hospital level>									
Hospital classification									
Teaching hospital or general hospital	1.000			1.000			1.000		
Hospital	1.078	0.845	1.373	0.929	0.706	1.223	1.005	0.839	1.205
Ownership									
Public	1.000			1.000			1.000		
Private	1.201	0.906	1.591	1.144	0.851	1.536	1.169	0.955	1.431
The number of beds									
Low	1.043	0.779	1.395	0.802	0.591	1.088	0.901	0.732	1.109
Middle	0.945	0.746	1.198	0.754	0.597	0.953	0.835	0.708	0.984
High	1.000			1.000			1.000		
The number of doctors per bed									
Q1	1.334	0.976	1.823	1.217	0.888	1.669	1.276	1.026	1.587
Q2	1.406	1.072	1.843	1.168	0.898	1.519	1.280	1.064	1.540
Q3	1.009	0.789	1.290	1.036	0.829	1.295	1.033	0.878	1.215
Q4	1.000			1.000			1.000		
MRI									
No	1.000			1.000			1.000		
Yes	1.182	0.935	1.495	1.154	0.883	1.508	1.157	0.971	1.379
Year									
2003	1.000			1.000			1.000		
2004	1.065	0.563	2.014	1.318	0.759	2.289	1.234	0.813	1.875
2005	1.544	0.885	2.694	1.115	0.653	1.905	1.352	0.919	1.990
2006	1.065	0.563	2.014	0.874	0.508	1.505	0.863	0.575	1.294
2007	1.544	0.885	2.694	1.043	0.622	1.749	1.027	0.699	1.510
2008	1.065	0.563	2.014	1.181	0.715	1.950	1.203	0.829	1.744
2009	1.544	0.885	2.694	0.895	0.534	1.500	0.943	0.644	1.380
2010	1.065	0.563	2.014	1.043	0.634	1.716	1.085	0.751	1.568
2011	1.544	0.885	2.694	0.938	0.566	1.552	1.000	0.691	1.447
2012	1.065	0.563	2.014	1.197	0.736	1.948	1.182	0.825	1.693
2013	1.544	0.885	2.694	1.125	0.689	1.838	1.032	0.717	1.484

*Abbreviations*: *COPD* chronic obstructive pulmonary disease, *ED* emergency department, *LOS* length of stay, *MRI* magnetic resonance imaging.

### Cox proportional hazard model for pneumonia-specific 30-day readmission associated with combined effects of number of doctors per bed and number of beds

**Fig. 2:**
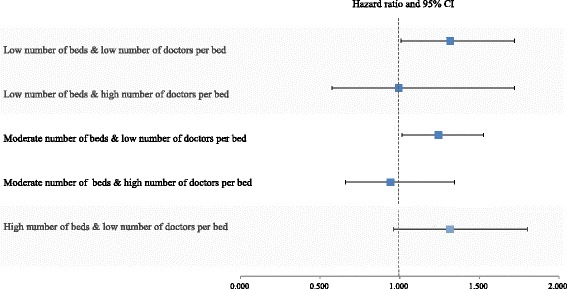
Hazard ratios and 95% confidence intervals (error bars) for pneumonia-specific 30-day readmission associated with the combined effect of number of doctors per bed and number of beds. Values are adjusted for patient and hospital characteristics

Figure 2 shows the results of a multivariate Cox proportional hazard model for pneumonia-specific 30-day readmission associated with the combined effect of the number of doctors per bed and number of beds. Pneumonia-specific 30-day readmission was significantly higher for patients admitted to hospitals with fewer beds and doctors per bed than those admitted to better equipped and staffed hospitals (HR = 1.317, 95% CI = 1.008–1.722). In addition, hospitals with a moderate number of beds and a lower number of doctors per bed were associated with pneumonia-specific 30-day readmission (HR = 1.243, 95% CI = 1.013–1.526).

## Discussion

This paper examined the effect of the number of doctors per bed on 30-day readmission, generally considered as an indicator of quality of care, particularly when patients are readmitted for the same condition due to treatment failure [19].

In this study, of the 7446 patients admitted for pneumonia, 19.1% were readmitted to the hospital within 30 days, and the pneumonia-specific readmission was 53.2%. In the U.S., the proportion of patients readmitted after pneumonia hospitalization within 30 days was 18.3% and readmission for the same condition was 22.4% [16]. The corresponding values in a study conducted in northern Spain were 7.3% and 35.8% [19]. Similarly, the 90-day readmission rate was 11.2% in Canada [21]. Compared to our data, pneumonia readmission within 30 days of discharge was on the high side in South Korea, and pneumonia-specific readmission was considerably higher than that of other countries.

We found that patients with pneumonia treated in hospitals with a higher number of doctors per bed were less likely to be readmitted. In addition, risk factors of pneumonia-specific readmission were different from those of pneumonia-unrelated readmission. Of interest, pneumonia-specific 30-day readmission showed significant relationships with the combined effect of number of doctors per bed and number of beds. Pneumonia patients admitted to hospitals with a smaller number of beds and a smaller number of doctors were more likely to be rehospitalized for the same condition. The 30-day pneumonia-specific readmission rate for patients admitted with pneumonia was explained better by the combined effect of physician and bed volumes than the number of doctors per bed alone.

It is unclear why a lower doctor staffing level is related to higher readmission rates. Fewer doctors may not be able to provide care for patients requiring additional attention by medical professionals. Alternatively, a lower number of doctors per bed may make it difficult to treat the complex medical conditions related to pneumonia.

The findings of this study are similar to those of previous investigations which showed that better outcome was associated with higher doctor staffing levels [22–24]. In case of socioeconomic status (SES) factors such as income, there are arguments about SES and readmission, although CMS does not adjust for SES when calculating readmission [25]. The findings of the current study that readmission was not related to income level was similar to those of other studies. However, in South Korea, pneumonia readmission rates were publicly reported for quality evaluation starting from 2015 [26]. Therefore, more studies about risk factors for pneumonia readmission are required to improve healthcare quality in Korea. Understanding the association between the number of doctors per bed and hospital readmission for pneumonia can provide insights into which hospitals are likely to show high readmission rates and how to prevent this outcome.

There are several limitations in this study. First, this study could not consider illness severity due to data limitation. An inaccurate severity control could act as a source of bias because severe patients tend to be admitted to tertiary or large hospitals. Second, we were unable to classify doctors by job specification because the data used in this study lacked information on the type of doctor. Third, although we used cohort data, we urge caution in interpreting the study results. This study demonstrated an association between the number of doctors per bed and readmission. However, this result does not prove causality. Fourth, because this study only included patients aged 65 years or older, results may differ from patients younger than 65 years of age. As pneumonia characteristics are very different between the elderly and the young, patients under 65 years of age were not included in this analysis.

Despite these limitations, this study has several strengths. First, we used national cohort sample data on health insurance claims, which includes patient demographic and socioeconomic data, medical history, and hospital information for about 1 million people. This representative nationwide sample ensured high external validity. Second, we adjusted for both patient- and hospital-level factors because both types affect readmission and ultimately, patient outcome. To our knowledge, most studies on 30-day readmission have focused on patient demographic and disease-specific factors. Third, we tried to design homogeneous groups and to focus on readmission due to in-hospital treatment failure by excluding readmission for other health problems in the subgroup analysis. Fourth, this paper provides valuable information for policy makers in managing readmission and improving quality of care. As hospital readmission rates can differ according to the number of doctors per bed, implementation of different types of strategies is required depending on the number of doctors in a specific hospital. Fifth, this study attempted to focus on unplanned and avoidable readmissions to assess quality of care and we only included readmissions for pneumonia to the same hospital.

## Conclusion

In summary, our results show that patients with pneumonia cared for by a higher volume of doctors were less likely to be readmitted. Notably, the pneumonia-specific 30-day readmission was significantly associated with the combined effect of physician and hospital bed volumes. Recently, Korea has focused on increasing National Health Insurance coverage and reducing medical costs. In the coming decades, Korea should focus on improving the quality of care [27]. The findings described here suggest that policy makers should continue to monitor readmission rates and select hospitals with a low number of doctors per bed to ensure quality improvement, particularly among hospitals with few doctors and many beds.

## Abbreviations

AMI: Acute myocardial infarction
CCI: Charlson comorbidity index
CMS: Centers for Medicare & Medicaid Services
COPD: Chronic Obstructive Pulmonary Disease
LOS: Length of stay
MRI: Magnetic resonance imaging
NHIS: National Health Insurance Service
OECD: Organization for Economic Cooperation and Development
SES: Socioeconomic status
